# Hard tissue formation in pulpotomized primary teeth in dogs with nanomaterials MCM-48 and MCM-48/hydroxyapatite: an in vivo animal study

**DOI:** 10.1186/s12903-024-04098-9

**Published:** 2024-03-11

**Authors:** Sahar Talebi, Nosrat Nourbakhsh, Ardeshir Talebi, Amir Abbas Nourbakhsh, Abbas Haghighat, Maziar Manshayi, Hamid Reza Bakhsheshi, Razieh Karimi, Rahman Nazeri, Kenneth J.D. Mackenzie

**Affiliations:** 1 Dentist, Research Committee, School of Dentistry, Isfahan University of Medical Sciences, Isfahan, Iran; 2 Department of Pediatric Dentistry, Dental Research Center, Dental Research Institute, School of Dentistry, Isfahan University of Medical Sciences, Isfahan, Iran; 3 Department of Pathology, Medical School, Dental Research Center, Dental Research Institute, Isfahan University of Medical Sciences, Isfahan, Iran; 4 Department of Materials Science, Shahreza Branch, Islamic Azad University, Shahreza, Iran; 5 Department of Maxillofacial Surgery, Dental Research Center, Dental Research Institute, School of Dentistry, Isfahan University of Medical Sciences, Isfahan, Iran; 6 DVM. Dental Science Research Center. Dentistry faculty, Isfahan University of Medical Sciences, Isfahan, Iran; 7 Advanced Materials Research Center, Department of Materials Engineering, Najafabad Branch, Islamic Azad University, Najafabad, Iran; 8 Dentist, School of Dentistry, Isfahan University of Medical Sciences, Isfahan, Iran; 9 MacDiarmid Institute for Advanced Materials and Nanotechnology, Victoria University of Wellington, Wellington, New Zealand

**Keywords:** Pulpotomy, Vital pulpotomy, Primary teeth, mineral trioxide aggregate, MCM-48, Hydroxyapatite, Hard tissue formation, Regeneration, Tooth preservation, Dental pulp, Dental pulp therapy, Animal study, Nano, Nanomaterial, Dental material, Puppies

## Abstract

**Background:**

This animal study sought to evaluate two novel nanomaterials for pulpotomy of primary teeth and assess the short-term pulpal response and hard tissue formation in dogs. The results were compared with mineral trioxide aggregate (MTA).

**Methods:**

This in vivo animal study on dogs evaluated 48 primary premolar teeth of 4 mongrel female dogs the age of 6–8 weeks, randomly divided into four groups (*n* = 12). The teeth underwent complete pulpotomy under general anesthesia. The pulp tissue was capped with MCM-48, MCM-48/Hydroxyapatite (HA), MTA (positive control), and gutta-percha (negative control), and the teeth were restored with intermediate restorative material (IRM) paste and amalgam. After 4–6 weeks, the teeth were extracted and histologically analyzed to assess the pulpal response to the pulpotomy agent.

**Results:**

The data were analyzed using the Kruskal‒Wallis, Fisher’s exact, Spearman’s, and Mann‒Whitney tests. The four groups were not significantly different regarding the severity of inflammation (*P* = 0.53), extent of inflammation (*P* = 0.72), necrosis (*P* = 0.361), severity of edema (*P* = 0.52), extent of edema (*P* = 0.06), or connective tissue formation (*P* = 0.064). A significant correlation was noted between the severity and extent of inflammation (*r* = 0.954, *P* < 0.001). The four groups were significantly different regarding the frequency of bone formation (*P* = 0.012), extent of connective tissue formation (*P* = 0.047), severity of congestion (*P* = 0.02), and extent of congestion (*P* = 0.01). No bone formation was noted in the gutta-percha group. The type of newly formed bone was not significantly different among the three experimental groups (*P* = 0.320).

**Conclusion:**

MCM-48 and MCM-48/HA are bioactive nanomaterials that may serve as alternatives for pulpotomy of primary teeth due to their ability to induce hard tissue formation. The MCM-48 and MCM-48/HA mesoporous silica nanomaterials have the potential to induce osteogenesis and tertiary (reparative) dentin formation.

**Supplementary Information:**

The online version contains supplementary material available at 10.1186/s12903-024-04098-9.

## Background

Currently, 3.5 billion people around the world are suffering from dental caries, one of the most common diseases. Notably, 60–90% of children are affected by this condition. Dental caries not only have a negative impact on the children’s quality of life but also impose substantial economic burdens on families, especially in cases where dental treatments must be performed under general anesthesia due to the child’s uncooperative behavior [1]. Despite advances in the prevention and treatment of dental caries, it remains a serious clinical problem, particularly in children, such that pulp therapy is among the most common dental procedures in pediatric dentistry [2]. Vital pulp therapy is a conventional procedure for the preservation of primary teeth with pulpal involvement. Pulpotomy is a well-accepted treatment procedure for the treatment of asymptomatic pulpal exposure in primary teeth. Initial diagnosis of radicular pulp status, preservation of pulp vitality, and adequate blood supply to the pulp are among the most important factors that play a role in the success of vital pulp therapy. The advent of new biomaterials that are biocompatible and have optimal sealability has opened up the possibility of revolutionizing the existing approaches to the preservation of pulp vitality in exposed primary teeth with reversible pulpitis.

According to the therapeutic goals, a pulpotomy medicament can be divided into categories of devitalization, preservation, and reparative and inductive regeneration [3]. Despite the excellent clinical success rate of formocresol in pulpotomy of primary teeth, the debate regarding its application has increased in recent decades following suggestions of its potential carcinogenesis and toxicity. Thus, attempts are ongoing to find a suitable alternative to formocresol for use in pediatric dentistry [4]. Formocresol is systemically absorbed and can trigger the humoral immune response. Moreover, it can result in the formation of hypoplastic lesions on permanent teeth and lead to pulp necrosis [5]. It can damage gingival tissue [6] and even lead to the development of dentigerous cysts [7]. Researchers suggest that biomaterials that can induce tissue regeneration by preodontoblasts can be the best alternatives to formocresol [8]. In other words, treatments aimed at regeneration are superior to other treatment modalities [4, 8, 9]. The currently suggested alternatives to formocresol include calcium hydroxide, ferric sulfate, mineral trioxide aggregate (MTA), freeze-dried bone, bone morphogenetic proteins, osteogenic protein, sodium hypochlorite, calcium-enriched mixture cement, enriched collagen solution, and synthetic nanocrystalline hydroxyapatite (HA) paste [3, 10]. The final goal of exposed pulp capping is to induce dentinogenesis by pulp cells. The ability to induce dentinal bridge formation is an important factor to consider in the selection of a pulpotomy agent [11–13].

At present, MTA is a standard material for apexogenesis, apexification (for infected immature teeth ), and root-end filling material [14]. It is also suitable for pulpotomy of primary teeth [15]. MTA induces the release of cytokines and can lead to hard tissue formation [16, 17]. A meta-analysis showed that MTA could be a good alternative to formocresol for the pulpotomy of primary teeth [18]. However, despite its advantages, MTA has some drawbacks, such as high cost, long setting time, difficult handling, potential tooth discoloration, and the possibility of leakage of lead, arsenic, and chromium [19, 20]. To overcome these shortcomings, other MTA-like materials, including bioactive glass, BioMTA, Biodentine, EndoSequence, and BioAggregate, were introduced to the market [20–22]. Despite some advantages, these materials are costly and not readily available [20].

Recently, researchers have attempted to utilize nanotechnology for synthesizing intelligent stimuli-responsive nanomaterials, which exhibit remarkable specificity and versatility as efficient drug-delivery vehicles in biomedical applications. These nanomaterials aim to induce the formation of nanocrystalline apatite, ultimately creating bone and dentin-like structures through the proliferation and differentiation of osteoblasts, enhancing the healing process [1, 23–25].

In modern dentistry, nanomaterials have revolutionized regenerative dentistry, transitioning from laboratory research to clinical applications. They play a crucial role in the treatment of bone and dental defects, contributing to the development of innovative biomimetic nanostructures for purposes such as cell regeneration, targeted treatment, diagnostics, imaging, and the production of dental materials [26]. All nano- and biomaterials utilized in dentistry have their respective flaws and advantages. Nanotechnology has played a crucial role in enhancing the quality of these materials and expanding their application scope. The introduction and application of nanoparticles have successfully addressed many of the initial drawbacks associated with these materials. Nanoparticles, including graphene, carbon nanotubes, hydroxyapatite, silver, silica, titania, zirconia, etc., contribute to elevating the quality of various bioproducts by incorporating diverse functional groups into them [27]. These chemical substances exist as individual units in small-sized particles, typically ranging from 1 to 100 nm, in an unbound state. This unbound state allows particles to form aggregates with one or more external dimensions, resulting in a high surface area. In restorative dentistry, nanomaterials are widely used in manufacturing nano-composite resins, bonding agents, endodontic sealants, coating materials, and bioceramics [26].

Nanomaterials have recently been used to load antimicrobial substances to prevent dental caries. In 2023, Xu Y et al. introduced a novel multidrug delivery system that provides intrinsic anti-caries and restorative properties in a bacteria-triggered manner to prevent dental caries [1].

MCM-48 is a type of mesoporous material that belongs to the family of Mobil Composition of Matter (MCM) materials. According to the International Union of Pure and Applied Chemistry (IUPAC) classification, the silica structure of MCM-48 and the composite of MCM-48 with hydroxyapatite (MCM-48/HA) are categorized as mesoporous materials with moderate porosity and pore diameters of 2–50 nm. Mesoporous materials with high surface areas and larger pore sizes are preferable to microporous materials (pore sizes < 2 nm) for entrapment [28–30].

MCM-48 is a highly ordered material with a regular arrangement of porous on the nanoscale. It was first synthesized and characterized by Chu et al. in the late 1990s [29]. Its three-dimensional cubic structure includes a network of cylindrical pores uniformly sized and distributed throughout the material. The unique characteristics of MCM-48, such as high surface area, large pore volume, and well-defined pore structure, make it attractive for various applications. It has been studied and used in areas such as catalysis, adsorption, separation, drug delivery, and nanotechnology. Additionally, MCM-48 has been investigated for drug delivery systems due to its ability to encapsulate and release drugs in a controlled manner. Its ordered pore structures can allow for precise control over drug release rates and improve drug stability. Some potential applications of MCM48 and MCM48/HA in medicine other than drug delivery are bone tissue engineering, Bio imaging, Bio sensing, Gene delivery, cancer treatment, and wound healing [28, 31–36].MCM-48/HA has shown promise in wound healing applications. The mesoporous structure of MCM-48 provides a favorable environment for cell attachment, proliferation, and migration, while the incorporation of hydroxyapatite promotes the regeneration of bone and soft tissue. MCM-48 /HA –based wound dressing can enhance the healing process and improve the overall outcome of wound treatment [28].

It is noteworthy that the application of family members of MCM nanomaterials in dentistry is an active area of research that holds potential for various applications in dentistry, such as dental Restorative materials [37], drug delivery in periodontal therapy [38, 39], dental implant coating [40], remineralizing agents [41, 42], local anesthetic delivery [43], and dental cement [44]. To the best of our knowledge, there is no study about the application of MCM-48 in the pulpotomy of teeth. Since the main goal of pulp therapy is to repair and regenerate the pulp tissue by dentinal bridge formation, this study aimed to synthesize two porous biomaterials, namely MCM-48 and MCM-48/HA, for pulpotomy of primary teeth and compare the pulpal response and hard tissue formation with the application of MTA in dogs.

## Materials and methods

### Synthesis of MCM-48

For this purpose, 2.4 g (6.6 mmol) of cetyltrimethylammonium bromide (CTAB) was dissolved in 50 mL of double-distilled water, and 50 mL of ethanol (0.87 mol) was added. The pH was adjusted to 12 with 12 mL of ammonium hydroxide (32 wt%, 0.20 mmol), and the solution was mixed at room temperature for 10 min using a magnetic stirrer before the addition of 3.4 g of tetraethyl orthosilicate (TEOS). After stirring for 2 h at room temperature, the resulting precipitate was filtered, rinsed with double-distilled water, and dried at room temperature. Unreacted CTAB was removed by calcination at 550 °C for 6 h in a crucible vessel, and the powder was ground in a mortar and pestle [30].

### Synthesis of MCM-48/HA

**Fig. 1:**
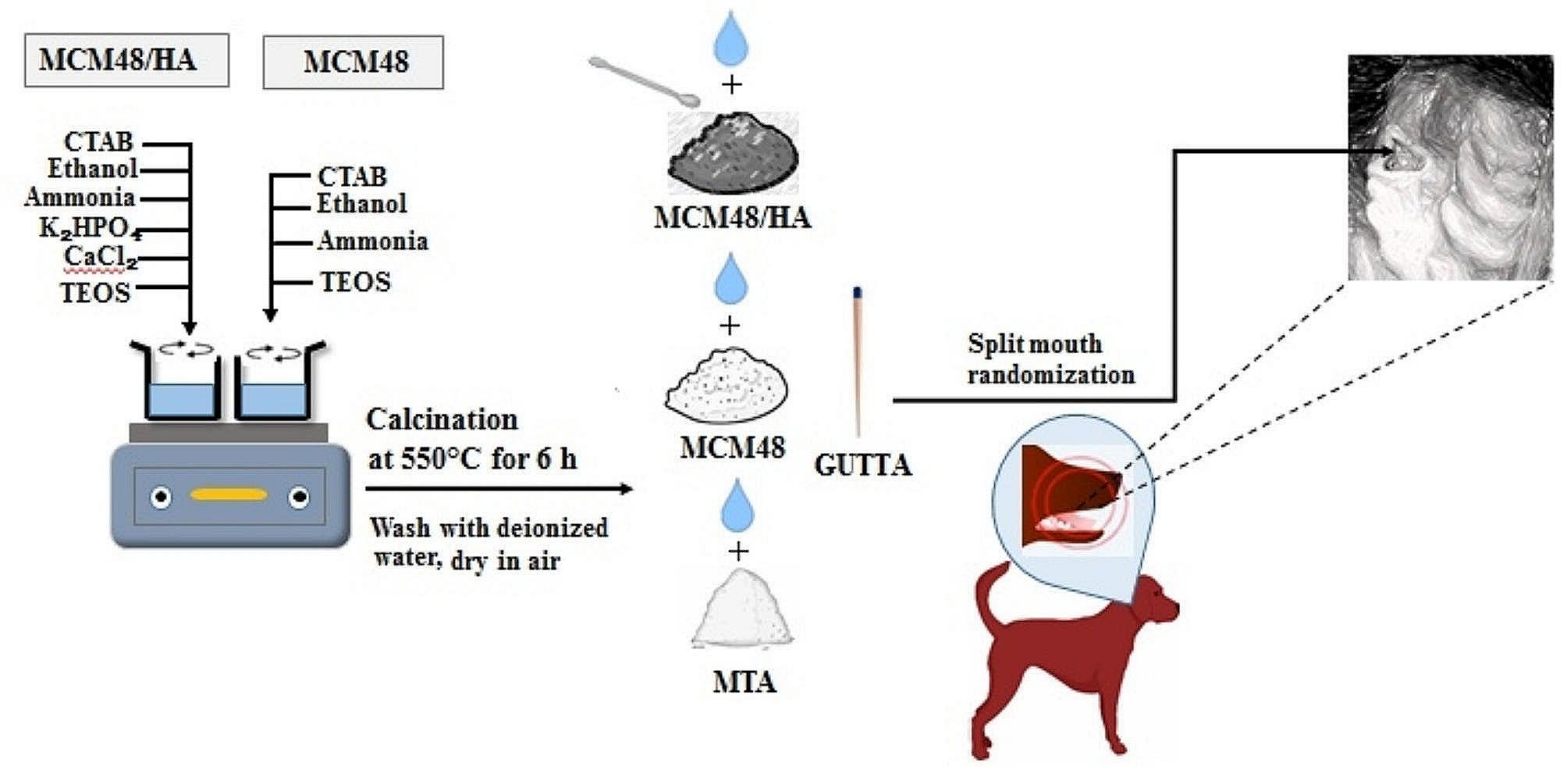
Schematic of methodology

For this purpose, 2.4 g (6.6 mmol) CTAB was dissolved in 50 mL of double-distilled water, and 50 mL of ethanol (0.87 mol) was added to produce a clear solution. Next, 0.4328 g (1.89 mol) of di-potassium hydrogen phosphate (K2HPO4) was added, and the pH was adjusted to 12 by the addition of 12 mL of ammonium hydroxide (32 wt%, 0.20 mmol). After stirring for 30 min, 1.042 g (9.48 mmol) of calcium chloride (CaCl2) was added, and the solution was stored at room temperature for 24 h due to the aging process. Following the addition of CaCl2 and K2HPO4 as the main components of hydroxyapatite, this aging process enhances the interaction between these components. The amount of calcium and phosphate in the solution was adjusted such that the Ca/P stoichiometric molar ratio was 5. Next, 3.4 g (16 mmol) of tetraethyl orthosilicate (TEOS) was added to the solution and stirred for 2 h at room temperature. The resulting precipitate was filtered, rinsed with double-distilled water, calcined at 550 °C for 6 h in a crucible vessel before being powdered in a mortar and pestle, and stored in autoclave-sterilized screw-top glass jars [30], as presented in Fig. 1.

### Microstructure characterization

A field emission scanning electron microscope (FESEM, Tescan, Mira 3 Czech Republic) was used to perform microstructural characterizations of the MCM-48 and MCM-48/HA samples. The instrument was equipped with an energy-dispersive X-ray spectroscopy detector (EDS, DXPeX10 P Digital X-ray Processor). Transmission electron microscopy (TEM; Phillips 208 m) was used to investigate the morphology of the synthesized powders. Using an X-ray diffractometer, the constituent phases were identified (XRD, D8 Advance, Germany). The experimental parameters for XRD were Cu–Kα X-ray radiation and a scan speed of 8° min^–1^.

### Animal model

The study design followed the animal study guidelines pertinent to the Isfahan University of Medical Sciences and ARRIVE and PRIASE 2021 Guidelines [45]. This study received approval from the Ethics Committee of Isfahan University of Medical Sciences (IR.MUI.REC.1395.3.576). The dogs were obtained from Dental Research Center at Isfahan University of Medical Sciences. This in vivo experimental study evaluated four female mongrel dogs aged between 6 and 8 and weighing 3–5 kg. The study was performed in accordance with the guidelines for the care and use of laboratory animals at Isfahan University of Medical Sciences. The inclusion criteria were dogs with 12 primary premolars in their mouth. The teeth had to be caries-free with no pathological lesions, such as abscess, swelling, nonphysiological mobility, or furcation involvement. The radiographic assessment revealed the formation of two-thirds of the root length, and they had no internal or external root resorption, furcation involvement, or periodontal ligament widening. The exclusion criteria were internal or external root resorption, furcation involvement, resorption of two-thirds of the root, periodontal ligament widening, dental caries, presence of pathological lesions such as abscess or swelling, nonphysiological mobility, and nonrestorable teeth (most commonly due to excessive wear).

### Split mouth randomization

Each tooth and each biomaterial were allocated a code. Two series of codes were used: the first series included #1 to #12 for the premolar teeth of the four quadrants (*n* = 12), and the second series included #1 to #4 for the random allocation of materials. The dogs had 12 primary premolar teeth (3 premolars in each quadrant). Thus, each biomaterial was used four times in the premolars of each dog. Allocation of the type of material to each tooth was determined by a blind investigator through drawing.

### Operation Procedure

**Fig. 2:**
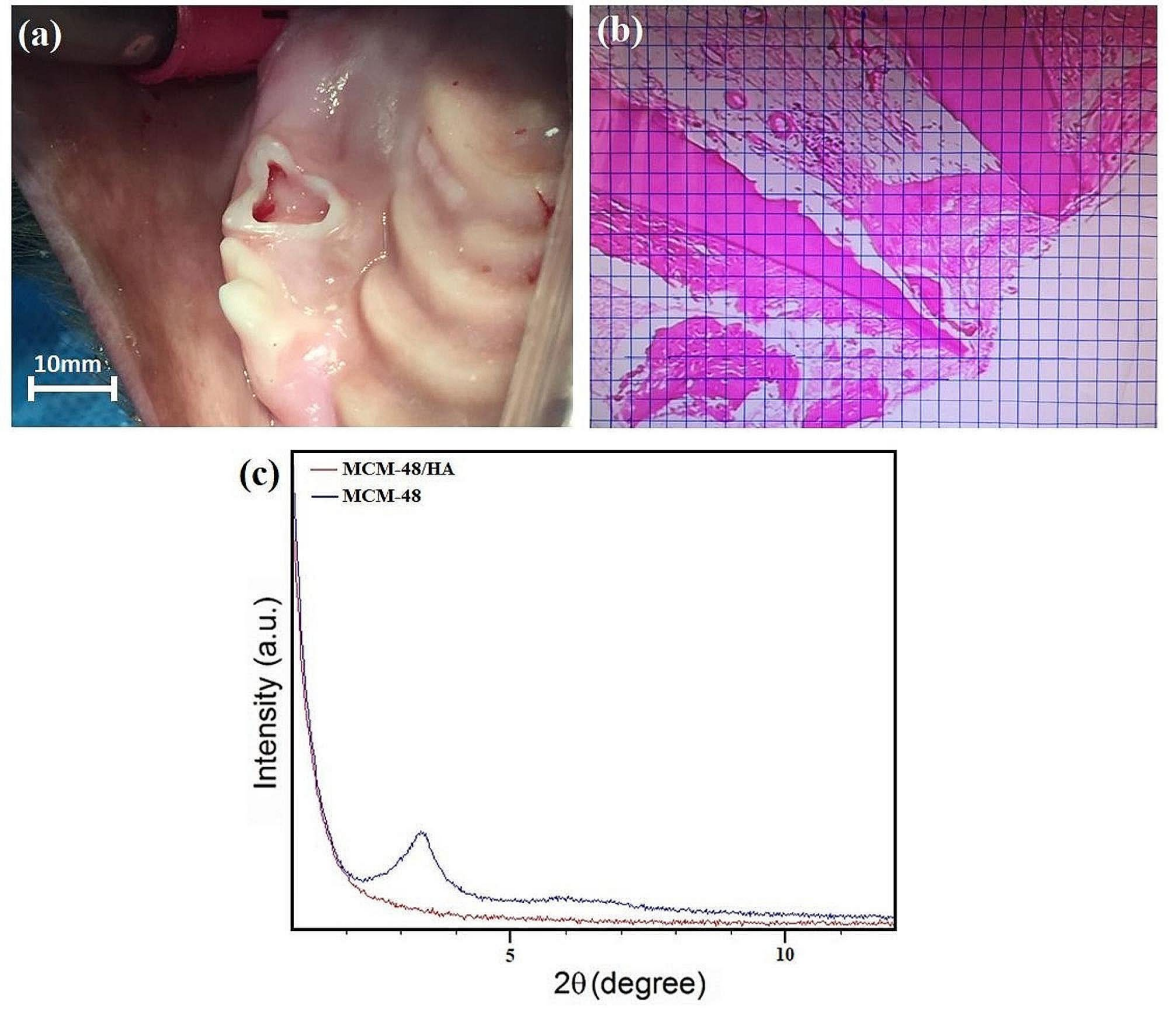
(**a**) Access cavity preparation, (**b**) calculation of the extent of osteogenesis and (**c**) powder XRD pattern of mesoporous MCM-48 and MCM-48/HA samples

Four female mongrel dogs, aged between 6 and 8 and weighing 3–5 kg, were kept in an individual room with controlled light and heat and access to food and water at the animal care unit of the Dental Research Center at Isfahan University of Medical Sciences. Since each dog had 12 primary premolars, a total of 48 teeth were evaluated. Each dog was anesthetized by subcutaneous injection of 0.02 mg/kg atropine (Alfasan, Holland) and intramuscular injection of 10 mg/kg ketamine hydrochloride (Alfasan, Holland) and 0.02 mL/kg 1% acepromazine (Alfasan, Holland). After intubation, anesthesia was continued with the administration of halothane (Nicholas, India). After taking a periapical radiograph by bisecting angle technique (Agfa, Primax, Berlin) from the respective tooth, the surrounding area was disinfected with 0.2% chlorhexidine (Iran Najo Pharmaceutical, Tehran, Iran). After calculus removal by ultrasonic scaler, a straight fissure bur (008) was used for access cavity preparation. Pulpal tissue was completely removed to the level of the cementoenamel junction by a sharp excavator, low-speed handpiece, and round bur. Next, hemostasis was induced at the canal orifice using a cotton pellet dipped in saline, as shown in Fig. 2a.

### Study groups

Depending on the group allocation of the tooth, the respective material was directly applied to the canal orifices as follows:

Group 1: MCM-48 was prepared by mixing the powder and liquid (sterile distilled water) in a 2:1 ratio and directly applied over the canal orifices using an amalgam carrier.

Group 2: MCM-48/HA was prepared as in group MCM-48 and directly applied over the canal orifices.

Group 3 (positive control group): white MTA (ProRoot; Dentsply, PA, USA) was mixed with distilled water according to the manufacturer’s instructions and applied over the canal orifices in paste-like consistency using the MTA carrier.

Group 4 (negative control group): gutta-percha (Orca; Diadent Europe B.V. Almere, Netherlands) was prepared in paste-like consistency and placed over the canal orifices as a neutral material.

In all groups, a 2 mm layer of intermediate restorative material (IRM; Type 3 Class 1; Dentsply, USA) was prepared in a paste-like consistency and applied over the materials. The teeth were finally restored with amalgam (SDI, Australia). In the MTA group, a sterile, moist cotton pellet was placed in the cavity 1 min prior to the application of IRM. Since it was not possible to postpone the final restoration for 1 week, which is routinely performed in pulp therapy with MTA in the clinical setting, the teeth were restored in the same session. All teeth underwent periapical radiography with the bisect angle technique for follow-up examinations. The same operator performed all treatments. Post-treatment radiographs were obtained after the procedures.

### Post-operative care after pulpotomy

After the procedure, all dogs received 5 mg/kg intravenous tramadol (Alborz Daroo, Iran) every 12 h for three days. For plaque control, 0.2% chlorhexidine was administered twice weekly until the second general anesthesia by a blind examiner to the study. Two and six weeks later, clinical examinations were performed by an experienced pediatric dentist, blinded to the treatment groups. A checklist was used to record the clinical success and failure criteria for assessing outcomes. The clinical success criteria included the absence of swelling, sinus tract, pathologic tooth mobility, and abscess. The presence of each of these symptoms was considered a clinical failure. The radiographic success criteria were: no evidence of internal or external resorption, furcation involvement, periodontal ligament widening, or peri radicular radiolucency. It should be noted that radiographic examination was carried out before tooth extraction to avoid additional general anesthesia, i.e., 4–6 weeks (depending on the time of natural exfoliation of primary teeth) after the pulpotomy procedure.

### Sample harvesting

the respective teeth were extracted traumatically via a mucoperiosteal flap under general anesthesia by an oral and maxillofacial surgeon, who were blind to the study, and the site was sutured using 3 − 0 black braided silk sutures (Supasil, Iran). The sutures were removed after one week. Dogs were given a soft diet for two days postoperatively.

### Post-operative care after teeth extraction

After the teeth extraction, all dogs received 30 mg/kg intravenous ampicillin (Jabber Ebn Hayyan, Iran) every 8 h for five days, 15 mg/kg intravenous metronidazole (Alborz Daroo, Iran) every 12 h for five days, and 5 mg/kg intravenous tramadol (Alborz Daroo, Iran) every 12 h for three days. After extracting the samples without the intention of sacrificing the animals, we allowed the dog’s permanent successor teeth to erupt until they reached seven months of age. Throughout this period, a veterinary surgeon conducted general health examinations to monitor any potential side effects resulting from the research. Additionally, a veterinary technician was responsible for their overall care. All animals were found to be free of disease, and their permanent teeth had fully erupted. Subsequently, the dogs were relocated from the research center and adopted by individuals associated with our team. The new owners were informed that the animals had been subjects of research for experimental materials.

### Histological Preparation

The teeth were individually placed in containers containing 10% buffered formalin. After 24 h, the specimens were immersed in 15% formic acid for decalcification. The acid was refreshed every three days. After one week, the softening (decalcification) of specimens was confirmed by checking with a needle [46]. The specimens were removed from the acid, coded, placed in a cassette, and subjected to 10% formalin twice (each time for 1 h) and 70%, 80%, 90%, 100%, and 100% alcohol in an orderly fashion, each for 1 h. They were then embedded in paraffin and sectioned into 3-µm sections using a microtome (Leica, Germany). The slides were then stained with hematoxylin and eosin and inspected under a light microscope (BX50; Olympus) at ×4, ×10 and ×40 magnifications. The inflammatory reactions and hard tissue formation were evaluated by a pathologist who was blind to the study. To quantify the histological findings and for comparison, each status was allocated a score. The scoring systems used in this study were presented by Stanley HR with some modifications [47] and are listed in Table 1.

**Table 1:** Pathologic reaction scoring system

**Severity of inflammation**		**Score**
No inflammation	Absence of neutrophils and lymphocytes	0
Insignificant inflammation	Scattered presence of neutrophils and lymphocytes (< 5%)	1
Mild inflammation	Mild presence of neutrophils and lymphocytes (5–20%)	2
Moderate inflammation	Moderate presence of neutrophils and lymphocytes (20–50%)	3
Severe inflammation	Severe presence of neutrophils and lymphocytes (> 50%)	4
**Connective tissue formation**		**Score**
Absence of connective tissue		0
Granulation tissue	Newly formed tissue comprising numerous capillaries in fine connective tissue and some inflammatory cells	1
Fibrotic tissue	Hyperplasia of fibroblasts along with increased collagen fibers	2
Combination of granulation tissue and fibrotic tissue		3
**Necrosis**		**Score**
Absence of necrosis		0
Presence of necrosis		1
**Severity of edema**		**Score**
No edema		0
Mild edema	0–30%	1
Moderate edema	30–60%	2
Severe edema	60–100%	3
**Severity of congestion**		**Score**
No congestion	1–3 capillaries	0
Mild congestion	3–6 capillaries	1
Moderate congestion	6–10 capillaries	2
Severe congestion		3
**Osteogenesis**		**Score**
Absence of newly formed bone		0
Presence of newly formed bone		1
**Quality of osteogenesis**		**Score**
Absence of newly formed bone		0
Calcified bone	Deposition of calcium and HA on fibrotic and necrotic tissues	1
Noncompact bone	Newly formed immature bone	2
Compact bone	With the Haversian system and mature bone structure	3
Dentin formation		4
**Extent of inflammation, connective tissue formation, necrosis, edema, and congestion**		**Score**
None		0
Lower third		1
Upper two-thirds		2
Entire tissue		3

Regarding the measurement of the extent of osteogenesis, a micrometer grid was used to quantify the surface area of the newly formed bone. For this purpose, 1 × 1 cm^2^ was drawn on a transparent sheet, and it was fixed to the monitor display with adhesive tape. Each square centimeter area served as one unit. The surface area of the hard tissue was quantified by counting the squares superimposed on the hard tissue. To convert each square centimeter unit on the 22-inch monitor to square micrometers and millimeters, the size of a red blood cell (7 μm) was used as the reference, meaning that each 1 cm side of a square was equal to 28 μm of the actual surface of the newly formed bone. Thus, each 1 cm^2^ unit of the monitor surface area would be equal to 784 µm^2^ or 0.000784 mm^2,^ as shown in Fig. 2b. To assess the total inflammation index, the extent of inflammation was multiplied by the severity of inflammation [48]. In addition to histological outcomes, clinical outcomes (swelling, dental abscess, sinus tract, and pathologic mobility) and radiographic outcomes (frequency of internal resorption, furcation radiolucency, periapical radiolucency, and external root resorption) were also evaluated and recorded.

### Statistical analysis

The sample size was calculated to be 12 in each group (4 dogs), considering 5% alpha and 80% power. The sample size was in accordance with previous animal studies as a base [49–51]. Data were analyzed using SPSS version 22 (SPSS Inc., IL, USA). The Kruskal‒Wallis test was applied to analyze the severity and extent of inflammation, congestion, edema, and quality of osteogenesis. Fisher’s exact test was used to analyze clinical examination (tooth mobility and swelling), radiography examination (internal and external root resorption, furcation, and periapical radiolucency), and histological examination for frequency of connective tissue formation, osteogenesis, necrosis and extent of connective tissue formation. Mann‒Whitney test was applied to analyze the extent of newly formed intra-group comparisons, and Spearman’s correlation coefficient was used for the correlation between severity and extent of inflammation. All tests were done at the *P* < 0.05 level of significance.

## Results

The low-angle XRD patterns of the MCM-48 and MCM-48/HA nano-composites are presented in Fig. 2c. The MCM-48 cubic la3d space group [52] is depicted to have exceptionally orderly structures. The peak at 2θ = 2–3° is missing in the MCM-48/HA specimens, presumably due to the MCM-48 pores being filled. The broad XRD traces reveal the existence of crystalline HA in all of the specimens, as well as CaO in MCM-48/HA specimens; this free CaO could play an important function in the formation stage. There is also a noticeable bulge in the baseline in the 2θ = 2–3° spectrum, especially in the composite specimens, which could be attributed to the existence of silica from MCM-48. It is noteworthy to mention that MCM-48/HA specimens possess specific surface areas (BET), pore diameters, and pore volumes of ^108 m2^/g, 2.12 nm, and 0.^31 cm3^/g, respectively. This implies that the pores of MCM-48 remain unfilled while free CaO and hydroxyapatite nanostructures form on the surface of MCM-48.

**Fig. 3:**
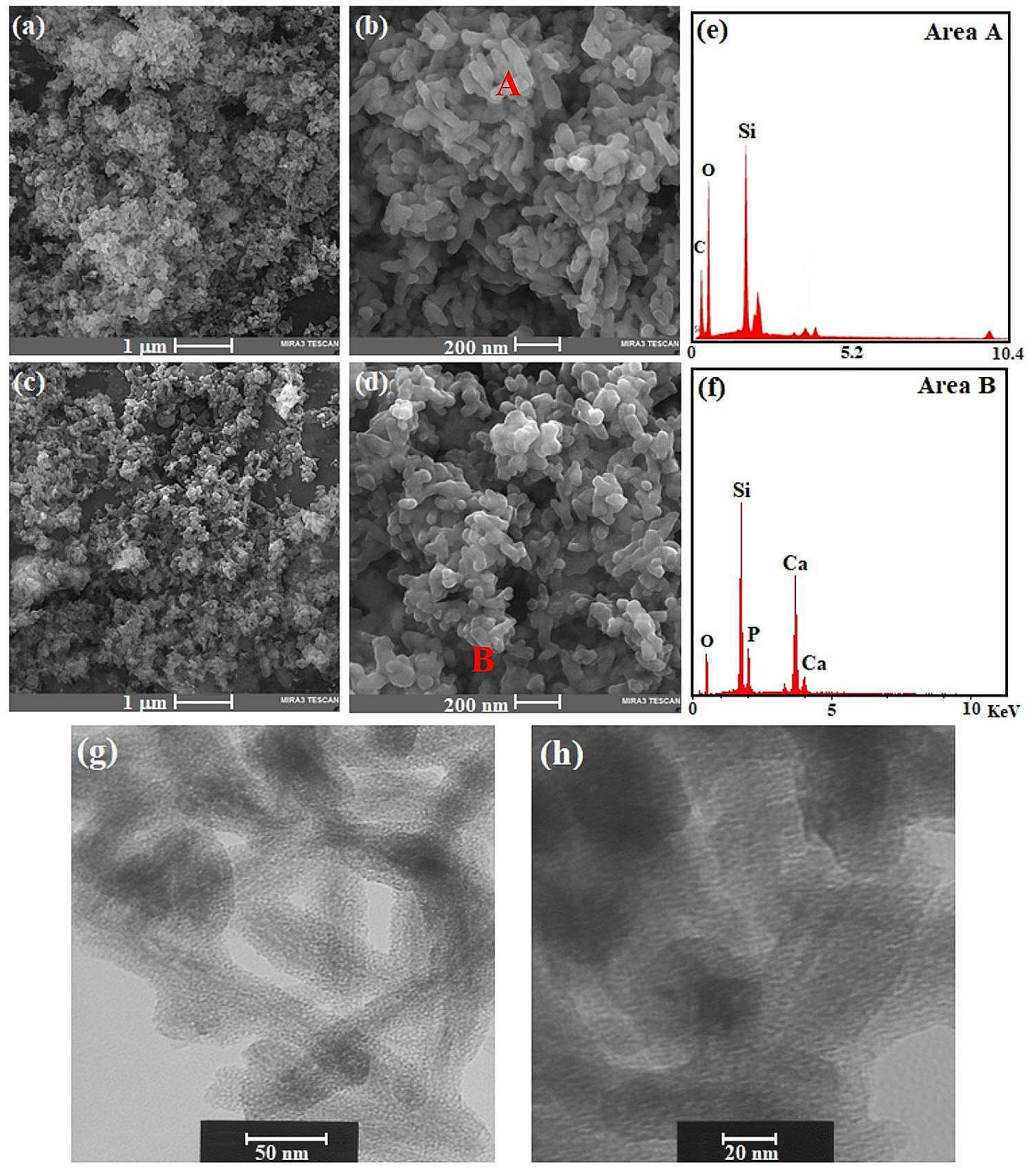
Scanning electron microscopy (SEM) photographs of (**a**, **b**) MCM-48 and (**c**, **d**) MCM-48/HA samples and (**e**, **f**) EDX analysis of areas A and B and (**g**, **h**) TEM images of the calcined MCM-48/HA sample

The MCM-48 and MCM-48/HA morphologies are depicted in Fig. 3a-d. MCM-48 and MCM-48/HA are tiny irregular particle agglomerations. Between the MCM-48 matrix and HA filler, there is almost no variation in the particle surface morphology. The high-magnification image shows that the clustered morphology of MCM-48/HA is substantially tangled together (Fig. S1). In FESEM micrographs, a variation in powder size was found for MCM-48 powder with an average value in the range of 50–120 nm, whereas particle sizes for MCM-48/HA powders were approximately 40–100 nm. The presence of O and Si (Area A) indicates the evolution of MCM-48; however, the existence of Ca, P, O, and Si within the crystallites of Area B in sample MCM-48/HA implies the existence of hydroxyapatite inside MCM-48 (Fig. 3e, f).

The pore diameter in sample MCM-48/HA is mostly approximately 2.2 nm, according to the TEM micrograph (Fig. 3g, h). This matches the BET results, implying that the pores are not entirely occupied. The calcinated MCM-48/HA sample has a morphology of semispherical particles that are uneven and fused, where the pore structure is largely evident.

### Clinical examination

In the MTA group, one of the 12 teeth was excluded from the study due to early exfoliation (*n* = 11). In the MCM-48 group, one of the teeth had one more root. Thus, 13 specimens were evaluated (*n* = 13). (It is important to highlight that asymmetrical root resorption was observed in all samples. We prioritized the root with the least resorption and greater evaluability. Notably, within the MCM-48 group, one tooth exhibited minimal resorption in both roots, making both accessible. Consequently, in this group, evaluation was conducted on both roots, leading to a total of *n* = 13). The two specimens in the gutta-percha group were excluded from histological analysis since no pulp tissue was observed (*n* = 10). At two weeks postoperatively, an abscess or sinus tract was not seen in any group. The swelling was noted in 1 specimen (8.3%) in the MCM-48/HA group. One specimen (8.3%) in the MTA group showed mobility. Fisher’s exact test revealed no significant difference in swelling and tooth mobility between the four groups at two weeks postoperatively (*P* = 0.735). At six weeks postoperatively, one tooth (8.3%) was exfoliated in the MTA group. Two teeth (15.4%) in the MCM-48 group and one tooth (8.3%) in the MCM-48/HA group showed edema. The four groups were not significantly different regarding swelling (*P* = 0.6) and exfoliation (*P* = 0.735).

### Radiographic examination

The four groups were not significantly different regarding the frequency of internal resorption (*P* = 0.168), furcal radiolucency (*P* = 0.695), periapical radiolucency (*P* = 0.602), or external root resorption (*P* = 0.99).

### Histological examination

**Fig. 4:**
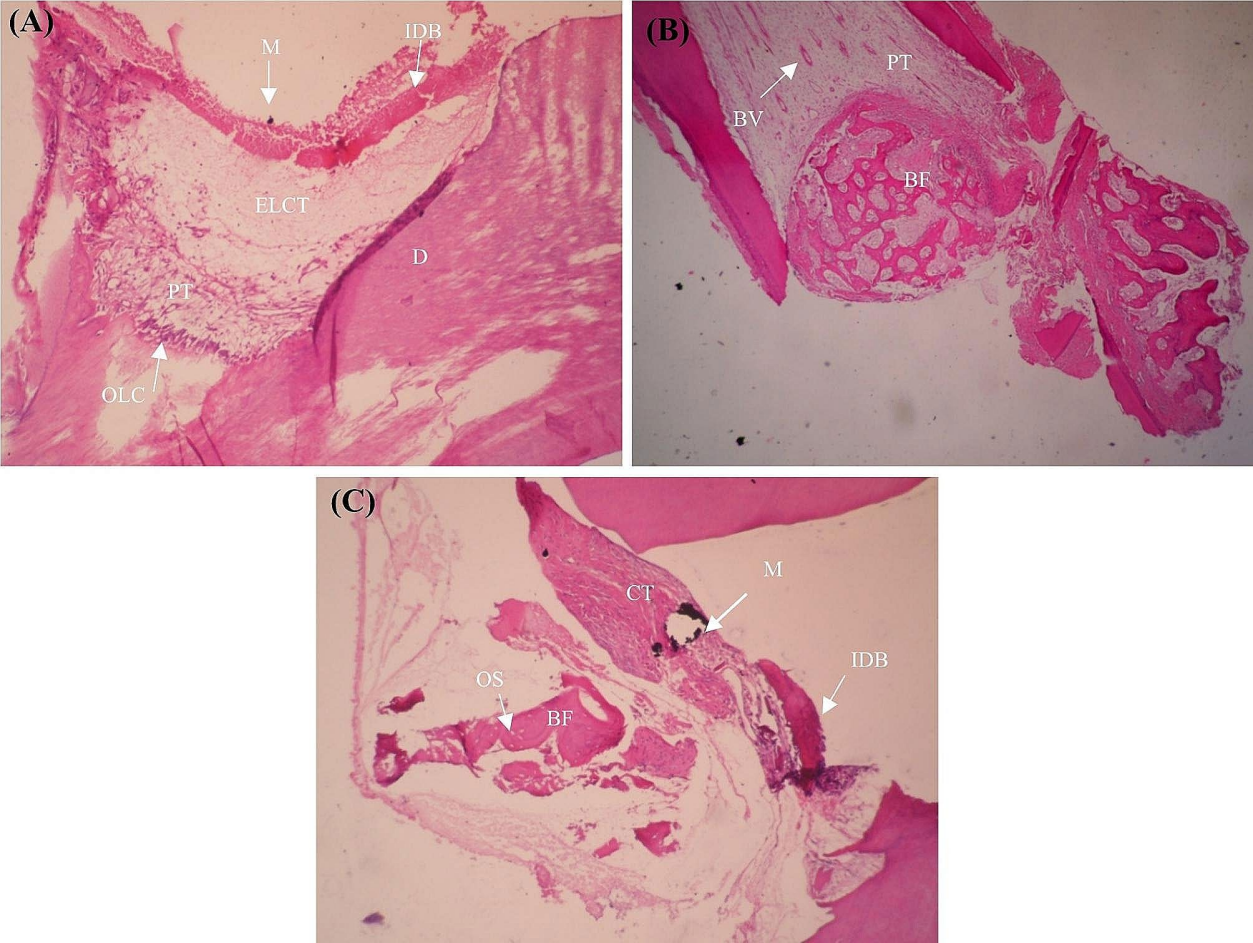
4 Photomicrographs of Hematoxylin and Eosin-stained MCM48-treated teeth. (**A**): interrupted dentin bridge (IDB), material (M), edematous loose connective tissue (ELCT), dentin (D), pulp tissue (PT), arrangement of odontoblast-like cells (OLC) ×10 magnification; (**B**): bone formation (BF), pulp tissue (PT), blood vessels (BV). ×4 magnification. (**C**): interrupted dentin bridge (IDB), material (M), connective tissue (CT), and lamellar bone formation(BF), including osteocytes (OS) in lacunas. ×10 magnification

Some degrees of inflammation were noted in all groups. The Kruskal‒Wallis test showed no significant difference in the severity (*P* = 0.53) or extent (*P* = 0.72) of inflammation between the four groups. Minimum inflammation was noted in the MCM-48/HA group, and maximum inflammation was noted in the MTA group (Table 2, Sect. 1). Pearson’s correlation coefficient showed a significant correlation between the severity and extent of inflammation (*r* = 0.954, *P* < 0.001). Since the total inflammation index was not normally distributed, the groups were compared with the Kruskal‒Wallis test, which revealed no significant difference in this respect between the groups (*P* = 0.56). Fisher’s exact test showed that the frequency of osteogenesis in the gutta-percha group was significantly lower than that in the other three groups (*P* = 0.012). However, the difference among the three other groups was not significant (*P* > 0.05). In other words, the gutta-percha group showed no osteogenesis (Fig. 4). After eliminating the gutta-percha group, the Kruskal‒Wallis test showed no significant difference in the quality of osteogenesis among the remaining three groups (*P* = 0.320, Table 3, Sect. 1). The surface area of the newly formed bone did not have a normal distribution. The Kruskal‒Wallis test showed a significant difference in this respect between the four groups (*P* = 0.03). The Mann‒Whitney test showed that the extent of newly formed bone in three groups was not significantly different (*P* > 0.05, Table 3, Sect. 2). Fisher’s exact test revealed no significant difference in the frequency of necrosis in the four groups (*P* = 0.361). The frequency of connective tissue formation was not significantly different among the four groups (*P* = 0.064). However, the extent of connective tissue formation was significantly different (*P* = 0.047, Table 2, Sect. 2). The Kruskal‒Wallis test found no significant difference in the severity of edema (*P* = 0.52) or its extent (*P* = 0.06) in the four groups. This test revealed significant differences in the severity of congestion (*P* = 0.02) and its extent (*P* = 0.01) between the four groups (Table 2, Sect. 3). Minimum congestion was noted in the MTA group, while maximum congestion was noted in the gutta-percha group.

**Table 2:** Frequency distribution of severity and extent of inflammation, connective tissue, and congestion

Section 1: Frequency distribution of severity and extent of inflammation in the four groups										
**Variable**		**Gutta-percha** (***N* = 10)**		**MTA** (***N* = 11)**		**MCM-48** (***N* = 13)**		**MCM-48/HA** (***N* = 12)**		**P value**
		Number	Percentage	Number	Percentage	Number	Percentage	Number	Percentage	
Severity of inflammation	No inflammation	6	60	6	54.5	8	61.5	9	75	0.53
	Insignificant	1	10	0	0	0	0	1	8.3	
	Mild	1	10	0	0	2	15.4	1	8.3	
	Moderate	1	10	0	0	1	7.7	0	0	
	Severe	1	10	5	45.5	2	15.4	1	8.3	
Extent of inflammation	None	6	60	6	54.5	8	61.5	9	75	0.72
	Lower third	0	0	2	18.2	1	7.7	1	8.33	
	Upper two-thirds	2	20	0	0	0	0	1	8.33	
	Entire tissue	2	20	3	27.3	4	30.8	1	8.33	
**Section 2.** Frequency distribution of connective tissue formation and its location in the four groups										
Connective tissue formation	Normal	8	80	9	81.8	6	46.2	2	16.7	0.064
	Granulation tissue	1	10	1	9.1	2	15.4	4	33.3	
	Fibrotic tissue	1	10	1	9.1	3	23.1	5	41.7	
	Granulation + fibrosis	0	0	0	0	2	15.4	1	8.3	
Extent of connective tissue	None	8	80	9	81.8	6	46.2	2	16.7	0.047
	Lower third	2	20	1	9.1	4	30.8	5	41.6	
	Upper two-thirds	0	0	0	0	1	7.7	3	25	
	Entire tissue	0	0	1	9.1	2	15.3	2	16.7	
**Section 3.** Frequency distribution of severity and extent of congestion in the four groups										
Severity of congestion	No congestion	3	30	10	90.9	5	38.5	8	66.6	0.02
	Mild	3	30	1	9.1	4	30.8	2	16.7	
	Moderate	4	40	0	0	3	23.1	2	16.7	
	Severe	0	0	0	0	1	7.7	0	0	
Extent of congestion	None	3	30	10	90.9	5	38.5	8	66.6	0.01
	Lower third	1	10	1	9.1	1	7.7	1	8.4	
	Upper two-thirds	0	0	0	0	4	30.7	0	0	
	Entire tissue	6	60	0	0	3	23.1	3	25	

**Table 3:** Frequency distribution and extent of osteogenesis and the quality of newly formed bone

**Section 1** Frequency distribution of osteogenesis and the quality of newly formed bone in the four groups										
**Variable**		**Gutta-percha** (***N* = 10)**		**MTA** (***N* = 11)**		**MCM-48** (***N* = 13)**		**MCM-48/HA** (***N* = 12)**		**P value**
		Number	Percentage	Number	Percentage	Number	Percentage	Number	Percentage	
Osteogenesis	Absence	10	100	6	54.5	5	38.5	6	50	0.012
	Presence	0	0	5	45.5	8	61.5	6	50	
Quality of formed bone	Calcified tissue	0	0	1	20	1	12.5	3	50	0.320
	None-compact bone	0	0	0	0	4	50	1	16.7	
	Compact bone	0	0	4	80	3	37.5	2	33.3	
**Section 2** Mean extent of newly formed bone in the four groups (based on the number of counted pixels)										
**Group**		**Mean**		**Std. deviation**		**Minimum**		**Maximum**		
GP		0		0		0		0		
MTA		9.2		21.6		0		74		
MCM-48		29.8		67.8		0		250		
MCM-48/HA		79.7		154.7		0		490		
P value		0.03								

**Fig. 5:**
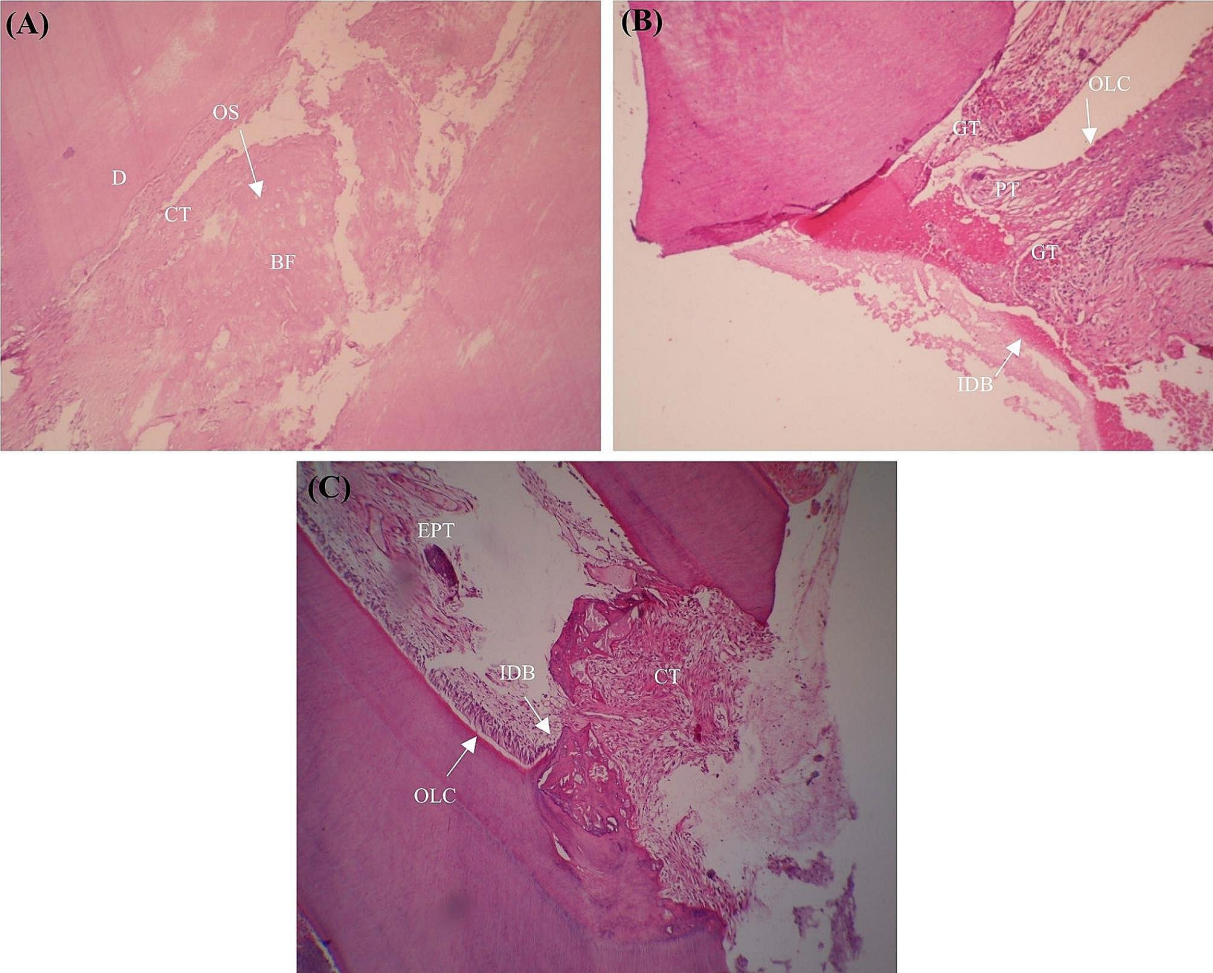
Photomicrographs of Hematoxylin and Eosin-stained MCM48-treated teeth. (**A**): interrupted dentin bridge (IDB), material (M), edematous loose connective tissue (ELCT), dentin (D), pulp tissue (PT), arrangement of odontoblast-like cells (OLC) ×10 magnification; (**B**): bone formation (BF), pulp tissue (PT), blood vessels (BV). ×4 magnification. (**C**): interrupted dentin bridge (IDB), material (M), connective tissue (CT), and lamellar bone formation(BF), including osteocytes (OS) in lacunas. ×10 magnification

**Fig. 6:**
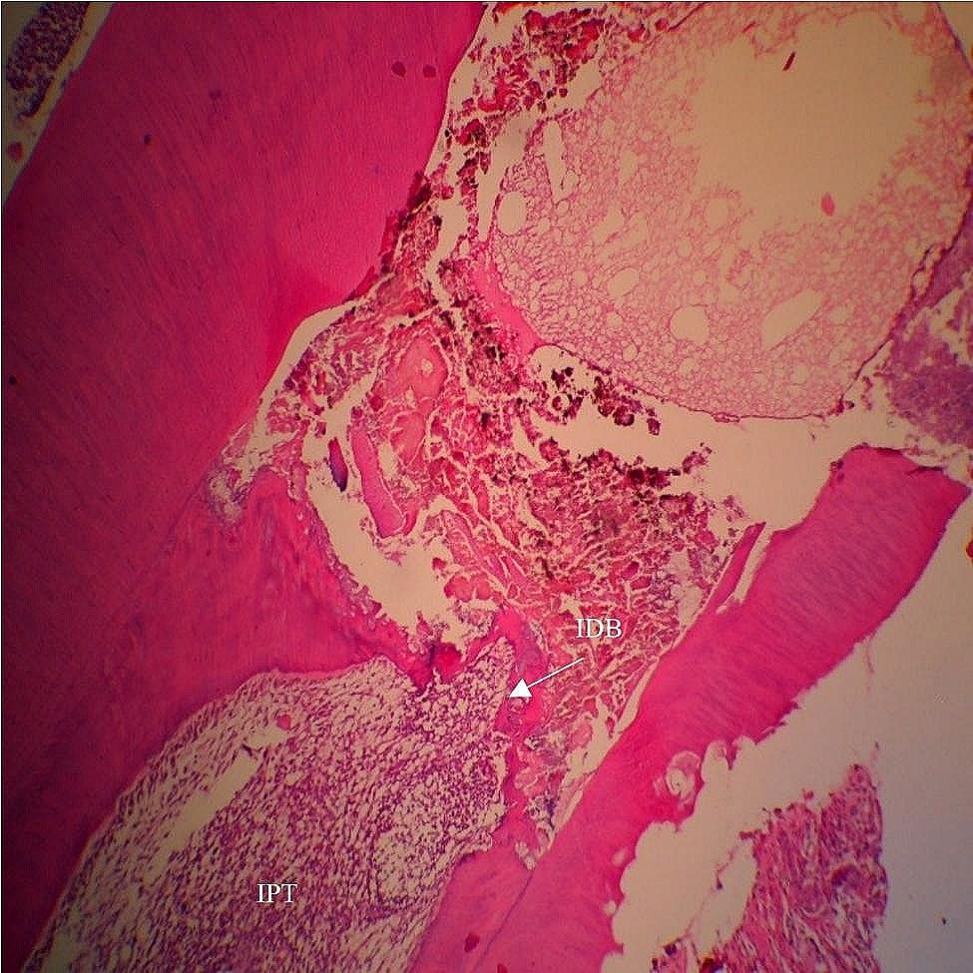
Photomicrographs of Hematoxylin and Eosin-stained MCM48/HA-treated teeth. Interrupted dentin bridge (IDB) and inflamed pulp tissue (IPT). ×10 magnification

**Fig. 7:**
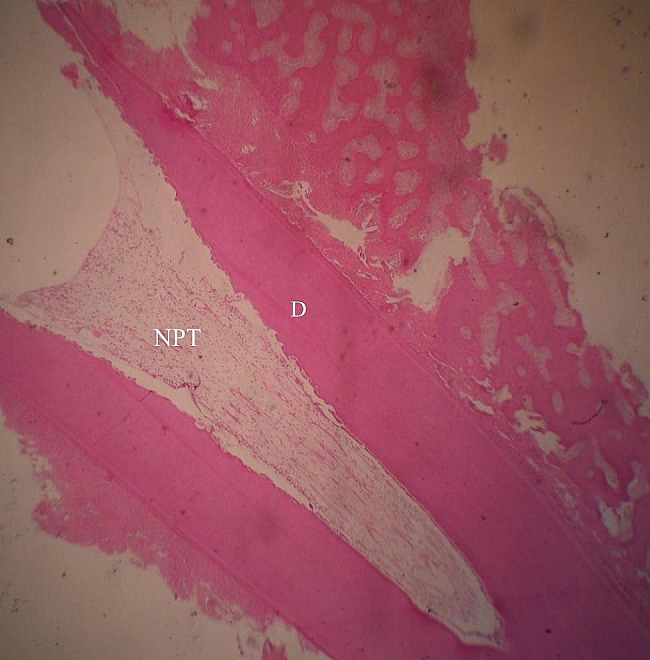
Photomicrographs of hematoxylin and eosin-stained MTA-treated teeth. Normal pulp tissue (NPT) and dentin (D). ×4 magnification

No significant difference was noted regarding bone quality among the remaining three groups after excluding the gutta-percha group. However, the maximum frequency of compact bone formation was noted in the MTA group. In terms of quantity, the mean amount of hard tissue formation was highest in the MTA group, followed by the MCM-48 and MCM-48/HA groups (Figs. 5, 6 and 7).

## Discussion

This in vivo study on dogs aimed to evaluate two novel nanomaterials for pulpotomy of primary teeth and assess the short-term pulpal response and hard tissue formation in comparison to the use of MTA under similar conditions. The results indicated that although the inflammatory reactions were variable in the groups of materials, no significant difference was noted in the severity or extent of inflammation between the groups. In total, 62.75% had no inflammation, and 37.25% had mild to severe inflammation. Inflammatory changes are the expected pulpal response to any extrinsic material.

Generally, the injury process respectively consists of: edema, congestion, inflammation, granulation tissue formation, fibrous tissue formation, and necrosis, which can involve one or several of these factors simultaneously. On the other hand, the healing process consists of the formation of fibrous connective tissue, calcium deposition on connective tissue, formation of hard tissue (non-dense or newly formed bone), formation of dense bone tissue, and formation of dentin, respectively [53]. In this study, we have modified the scoring system in a way that allows for the evaluation of all the above-mentioned processes. In the present study, minimum inflammation was noted in the MCM-48/HA group, and maximum inflammation was noted in the MTA group, with no significant difference. Evidence shows that extrinsic materials can induce inflammation early after their application; however, the severity of inflammation subsides over time [54]. Regarding connective tissue formation, maximum normal tissue was noted in the gutta-percha and MTA groups, while minimum tissue formation was noted in the MCM-48/HA group. Histologically, maximum granulation and fibrotic tissues were noted in the MCM-48/HA group. From a histological perspective, considering the formation of fibrous connective tissue in healing subsequent to granulation tissue can act as a double-edged sword and play a positive and negative role in the healing process. However, if interpreted in fibrosis or scar tissue, it would have a negative and detrimental implication [53]. Indeed, it is interesting to note that the bone is one of the few tissues that can be healed without forming a fibrous scar [55]. There is evidence that deposition of calcium or HA may occur on disorganized connective tissue, and in favorable conditions, this may lead to compact bone or dentin bridge formation [50]. In this study, edema was evaluated separately from other reactions because some cases showed extensive, severe edema of the pulp with no other pathological symptoms (such as inflammation, necrosis, or congestion). Minimum and maximum frequencies of congestion were noted in the MTA and MCM-48 groups, respectively. Congestion refers to the dilation of capillaries and the accumulation of red blood cells. Amputation of coronal pulp in cervical pulpotomy leads to vascular changes in the pulp and the formation of a capillary network around the excavated area. After two weeks, this area becomes shallower, and a flat capillary network forms (when the slight formation of the dentinal bridge is noted). After 4 weeks, the thickness of the dentin bridge increases, and an underlying capillary network form. After 8 weeks, the vascular network beneath the dentinal bridge resembles a normal pulp. In bone defects, new bone formation with a trabecular pattern, similar to that of natural teeth, occurs within 3–6 weeks. This indicates that the same process takes place in teeth. Therefore, the selection of the 4-6-week study period in this investigation is justified for studying hard tissue formation [51].

In the current study, a maximum percentage of necrosis was noted in the MTA group, with no significant difference. Dominguez et al. [51], in their study on pulpotomy of dog teeth, reported that MTA triggered minimal pulpal inflammation, and pulpal necrosis occurred in 10% of the cases.

In terms of the quantity of osteogenesis, no osteogenesis occurred in the gutta-percha group, which is a significantly different outcome compared to the other groups. This result was anticipated due to the inert nature of gutta-percha. While gutta-percha is biocompatible, it is not bioactive and cannot induce osteogenesis. Gutta-percha was considered as the negative control. Considering that the main purpose of this study was to investigate the hard tissue formation ability of the tested materials, it seems that the selection of gutta-percha as the negative control group was correct.

It has been well-established that dental pulp possesses the ability to form a barrier of hard tissue (dentin bridge) following direct pulp capping or pulpotomy.

During reparative dentinogenesis, primary odontoblasts in the exposure site undergo destruction and are replaced by differentiated odontoblast-like cells. In pulp wound healing, migration of precursor cells and stem cells to the injured site, their proliferation, and differentiation into odontoblast-like cells occur. Reparative dentinogenesis often begins with the formation of a fibrodentin matrix, which is atubular and irregular, accompanied by cuboidal-shaped cells. The initiation involves the formation of a tubular dentin-like matrix, elongation, and polarization of odontoblast-like cells. Despite extensive studies, the signaling molecules involved in cellular differentiation during dentinogenesis are not yet well understood. It seems that fibronectin, which is an extracellular matrix glycoprotein along with the dentin matrix, plays a fundamental role in the final differentiation of odontoblasts. On the other hand, during reparative dentinogenesis, when the dentin epithelial basement membrane is absent, the adhesion of precursor cells to a suitable surface (predentin) is likely a crucial prerequisite for the differentiation of cells forming hard tissue [56].

As we know, the stem cells present in the pulp are located in the perivascular environment around the pulp vessels. Suppose the pulp stimulus is mild or gradual. In that case, odontoblasts are usually viable, and they can form a dentin barrier under the influence of the injury, allowing the underlying pulp tissue to maintain its function. The essential strategy in these cases is the preservation of the remaining odontoblasts. However, when the stimulus is moderate or rapidly progressing, such as in the case of severe decay or fracture, the primary odontoblasts will undergo destruction. However, the chemical structure of the tissue barrier formed in response to materials used in Vital Pulp Therapy (VPT) has been somewhat limitedly determined. Whether the composition of the barrier components is similar to that of healthy dentin or not remains unknown. Based on this, researchers have suggested conducting additional studies not only in chemical analysis but also in the areas of Immunohistochemistry (IHC) and electron microscopy on the tissue barrier [51].

Regardless of the type of tissue reactions, the intrinsic properties of the tested materials also vary. Regarding the formation of hard tissue, the mechanisms in MTA, MCM-48, and MCM-48/HA differ from each other. The mechanism of hard tissue formation following the use of MTA in pulp capping is similar to calcium hydroxide. Calcium oxide, as one of the main components, transforms into calcium hydroxide when mixed with powder and water. Upon contact with tissue fluid, calcium ions react with carbon dioxide present in tissues, forming calcite microcrystals. Subsequently, fibronectin accumulates inside these particles, leading to cellular adhesion and differentiation. MTA is inherently devoid of phosphorus, and in order to form apatite crystals, it needs to be in proximity to phosphorus-containing solutions such as phosphate buffer saline (PBS). Carbonated apatite is characteristic of biological apatite phases in bone, cementum, and dentin, and it likely plays a reinforcing role in the dentinogenic activity of MTA [57]. The biocompatibility and bioactivity of MTA are primarily attributed to its ability to form apatite. The bioactivity of MTA is the factor responsible for its biocompatibility, dentinogenesis, and sealing ability [58].

As no previous study has assessed the efficacy of MCM-48 and MCM-48/HA as pulpotomy agents, the mechanism of hard tissue formation following the use of the material is unknown. However, the specific properties of MCM-48 and MCM-48/HA materials, particularly MCM-48/HA, are such that they release significant amounts of calcium ions (effective ions in the healing process), leading to an alkaline environment. Alkalinity is a prerequisite for wound healing. It has also been demonstrated that the substantial release of calcium is essential for stimulating apatite formation and the development of a dentin barrier [59]. In materials science, MCM-48/HA is a ceramic nano-composite. Generally, nano-hydroxyapatite structurally resembles natural bone and can, through inducing the formation of apatite crystals similar to the mineral phase, create a surface similar to natural bone. In other words, this material is not only bioactive but can also stimulate the proliferation and differentiation of osteoblasts, thus accelerating the healing process. Mesoporous materials based on silica are considered an excellent choice for tissue engineering [60]. Regular mesoporous materials can be used as scaffolds in tissue engineering. This localized delivery capability, coupled with bioactive behavior, has opened new horizons in the field of biotechnology [61]. The current study showed that both biomaterials successfully induced hard tissue formation. MCM-48/HA contains calcium and phosphorous ions, and the HA in its composition has a boosting effect. This may explain the high rate of hard tissue formation in this group. One of the key mechanisms by which MCM48 nanomaterial supports hard tissue formation is through its unique porous structure. The mesoporous nature of MCM48 provides a high surface area and pore volume, facilitating increased cell adhesion, proliferation, and migration. Moreover, the pores can be loaded with bioactive molecules, growth factors, or signaling molecules that can further enhance cellular response and contribute to the tissue regeneration process [61].

Drug delivery systems utilizing biocompatible materials play a crucial role in releasing drugs into target tissues within the oral cavity, minimizing side effects. Consequently, scientists have explored diverse delivery systems to enhance the effectiveness and acceptance of therapeutic approaches for dental issues and oral diseases. Additionally, biomaterials can be employed as carriers in biocompatible drug delivery systems [62]. As mentioned earlier, due to the unique property of drug release potential in MCM48 and MCM48/HA, medications can be loaded onto these mesoporous materials for extended slow release. In endodontic treatment, there is sometimes a necessity to utilize systemic antibiotics, analgesics, and anti-inflammatory agents. Considering the potential of targeted drug release from nanomesopores, the addition of very small amounts of the drugs mentioned above locally represents an innovation in endodontic treatments. Further studies are required to thoroughly explore this potential in dentistry.

We previously assessed the biocompatibility of MCM-48, MCM-48/HA, and MTA MTA (Proroot) with human gingival fibroblasts using the methyl thiazolyl tetrazolium (MTT) assay over different time intervals—28 h, 22 h, and two days—to varying concentrations of 0.2, 0.02, and 0.002 gr/µl. Our findings revealed that MCM-48/HA exhibited minimal cytotoxicity, maintaining a Ca/*P* = 5 stoichiometric ratio (the data has not been published). Additionally, we reported a 74% shorter setting time for MCM-48 compared to MTA. The final setting time for MCM-48/HA averaged 35 min, in contrast to MTA’s 135 min. Also, we found that these two materials were easy to handle [44].

Animal studies are widely used in dentistry, particularly in endodontology, where the exclusion of confounding human variables is necessary. This aids in comprehending a variety of biological and molecular mechanisms related to infection, disinfection, inflammation, necrosis, healing, regeneration, and disease progression before embarking on human clinical trials. Based on ethics and laws, animal studies are also essential for testing the safety and effectiveness of new dental materials to minimize health risks for humans. However, the translation of research observations from animal studies to humans has always been challenging. Animal studies are arguably amongst the most criticized, controversial, complex, challenging, expensive, labor-intensive, and highly regulated of all the types of studies within endodontology [45, 63, 64].

Animals utilized in previous studies as models for human pulpitis include rodents, rabbits, ferrets, swine, dogs, and non-human primates. The selection of the dog as a model is based on various criteria in animal selection strategies for pulp inflammation studies, such as the ease of performing procedures, proximity to human dental anatomy, knowledge about the model, and partial phylogenetic distance. Therefore, the dog is considered a suitable model for the present study [65].

In addition to histological assessment, clinical and radiographic examinations were also performed in this study, which revealed no significant difference between the groups regarding swelling and early exfoliation of teeth. Sinus tract and furcation involvement were not observed. The groups were not significantly different regarding radiographic signs and symptoms, which was in agreement with the results of Nourbakhsh et al. [66]. In this study, the absence of signs such as sinus tract, swelling, abscess, and pathologic mobility was considered a clinical success, and absence of furcation involvement, periapical lesion, and internal and external root resorption were considered radiographic success criteria [67]. In general, in short-term studies, the frequency of clinically and radiographically successful cases is higher than that of histologically successful cases. According to clinical and radiographic findings, all four materials in this study were successful. However, histologically, only 25 out of 48 teeth had normal pulp tissue.

Although all biomaterials in this study showed optimal biological properties, it should be noted that this study was conducted on sound, caries-free teeth, while in the clinical setting, carious teeth undergo pulpotomy. Thus, clinical studies are needed on carious teeth. Another finding of this study was the leakage of pulpotomy materials into the pulp, which can be due to the material composition or the application technique. The pulpotomy agent should be applied gently over the exposed pulp surface and should not be condensed. Leakage of material into the pulp tissue may decrease the rate of recovery and compromise dentin bridge formation [51]. An ideal pulpotomy agent should preserve healthy radicular pulp tissue, be nontoxic to the pulp and periapical tissue, be biocompatible and affordable, and not interfere with the natural process of root resorption. It should also have the potential to induce hard tissue formation [50].

This animal study, reported according to the ARRIVE and PRIASE 2021 Guidelines [45], faced some limitations, some of which are mentioned here.

Teeth isolation by rubber dam was not used in this study due to challenges in its applying to dogs’ teeth. Puppies have premolar teeth that differ somewhat in morphology compared to human primary molar teeth. We tried the wedge-it technique and split-mouth isolation, but both were unsuccessful in achieving proper isolation due to spaces between posterior teeth. While the use of rubber dam in animal studies allows for better standardization of the experimental conditions, it is important to note that its application in veterinary dentistry, especially in animal studies, may vary depending on specific research objectives, animal species, and the availability of suitable equipment.

However, this study was conducted under general anesthesia with meticulous control over the dry conditions of the working environment. Isolation was achieved with careful use of cotton rolls, gauze, a saliva ejector, powerful suction, cheek retractors, and a tongue guard. Also, the conditions were the same and standardized for all samples. Another limitation was taking radiographs. X-ray radiographs were essential for assessing success or failure based on radiographic criteria. Because obtaining intraoral radiographs for dogs is performed under general anesthesia, the possibility of conducting biweekly examinations, similar to clinical check-ups, did not exist to prevent the additional and unnecessary anesthesia. The bisecting technique was employed for obtaining radiographs, although determining the bisecting angle posed some difficulty. Disposable wooden sticks were utilized to facilitate the determination process.

Puppies are often highly active chewers and fractured primary teeth are common in dogs. Therefore, the proper selection of restoration material is crucial. While the standard restoration for pulpotomy of primary teeth in humans is Stainless Steel Crowns (SSC), using them in animal studies is practically unfeasible due to their design for human teeth. In similar animal studies, various materials such as composite, glass ionomer, and amalgam have been utilized. Due to our previous experience, where the use of glass ionomer for final restoration resulted in failure in some samples, we decided to use amalgam. On the other hand, achieving a retentive and resistant form for amalgam restoration was challenging. So, we opted to use IRM and amalgam for several reasons. Firstly, when a full pulpotomy is performed, a significant portion of the tooth crown is removed, and amalgam is preferable over other tooth-colored restorations from the point of durability. Secondly, Amalgam is a commonly used dental filling material in veterinary dentistry, known for its strength and durability, making it suitable for larger restorations or areas experiencing significant stress. On the other hand, in our previous study, glass ionomer was used to restore dogs’ teeth following cervical pulpotomy. However, the same rate of restoration loss occurred as observed in this study [66]. Based on our prior experience, glass ionomer restorations had failed in some samples. Additionally, the biocompatibility of Resin-Modified Glass Ionomer Cements (RMGIC) and traditional Glass Ionomer Cements (GIC) is compromised as HEMA may leach out from the RMGIC within the first 24 h of application [68]. Consequently, in this study, we opted to use amalgam and IRM. This choice allows for the contingency that if the amalgam restoration were to fail, the IRM would remain intact. Amalgam restoration of 8 teeth in the gutta-percha group, five teeth in the MTA group, six teeth in the MCM-48 group, and seven teeth in the MCM-48/HA group was partially or completely lost; however, at the end of the study, IRM remained intact in all samples. Moreover, approximately 63% of the samples had no inflammation, which implies that despite the failure of amalgam restorations in some samples, IRM material has demonstrated the ability to provide proper sealing of the access cavity.

Teeth restored in the MTA group were treated in a one-visit manner. Practically, in animal studies, waiting for the final setting of MTA is not possible. So, it is one of the serious limitations of using MTA in animal studies. Nevertheless, based on the outcomes of previous studies, it was hypothesized that pulp moisture would be adequate for the final setting of MTA.

In this study, we did not stain the specimens for possible bacterial contamination. Identifying bacteria within dental tissue is a complex procedure that requires specific protocols. While the Brown-Brenn method is widely employed for detecting bacteria in tissue sections, it does not consistently stain Gram-negative bacteria [69]. Eldeniz AU et al. evaluated the antibacterial activity of leachable components from various root-end filling materials, including amalgam, ProRoot MTA, IRM, Super Bond, Geristore, Dyract, and Clearfil APX composite with SE bond. They discovered that IRM and MTA exhibited generally more potent inhibitory effects on bacterial growth compared to the other tested materials, and they demonstrated optimal sealability [70].

Finally, the lifespan of primary teeth in puppies is short and varies. They typically begin erupting around 3–4 weeks of age and start to exfoliate around 12–16 weeks of age, making their lifespan shorter. Although it would have been preferable to harvest the samples at different time intervals in this study, the short lifespan of puppies’ teeth and the limitations in utilizing a larger number of animals made this impractical.

Despite these limitations, the current research results demonstrate that two nanomaterials, MCM-48 and MCM-48/HA, are capable of hard tissue formation.

## Conclusions

The present study suggests that MCM-48 and MCM-48/HA nanomaterials could serve as alternatives to MTA to pulp capping after pulpotomy. Both MCM-48 and MCM-48/HA nanomaterials have the potential to induce hard tissue formation and osteogenesis. The antibacterial activity, sealing ability, and mechanical properties of MCM-48/HA should be evaluated in future studies. Clinical studies are finally needed to confirm the results of animal studies.

### Electronic supplementary material

Below is the link to the electronic supplementary material.

Supplementary Material 1

## Data Availability

The datasets used and/or analyzed during the current study are available from the corresponding author upon reasonable request.
