# Supplementary figures and images for: Hard tissue formation in pulpotomized primary teeth in dogs with nanomaterials MCM-48 and MCM-48/hydroxyapatite: an in vivo animal study
Source: BMC Oral Health. 2024 Mar 11;24:322. doi: 10.1186/s12903-024-04098-9 (PMC10926592; doi:10.1186/s12903-024-04098-9)

**Supporting Information**


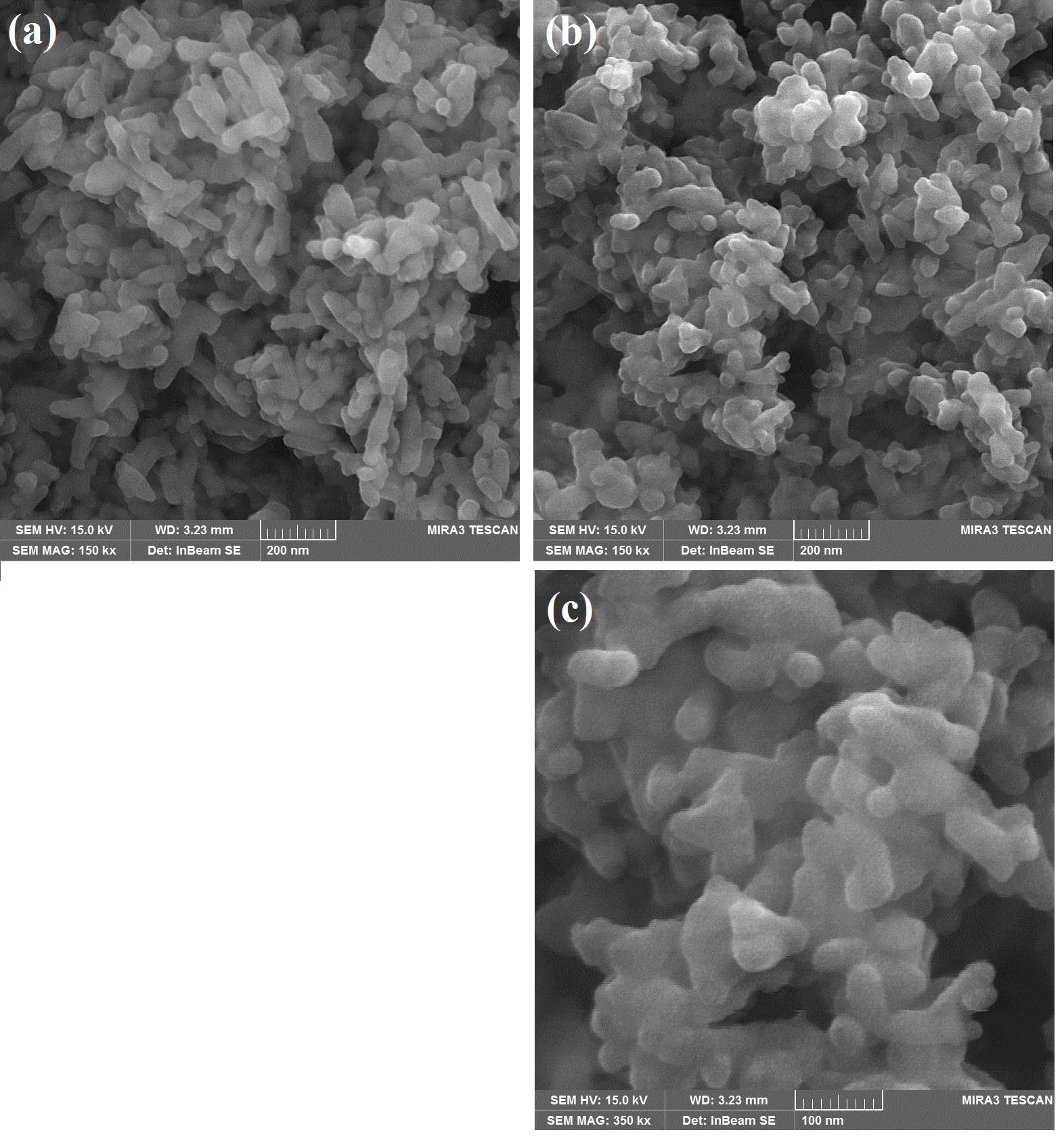


**Fig. S1. FESEM image of (a) MCM-48 and (b,c) MCM-48/HA samples**

Supplement: Supplementary file 1 — Supplementary Material 1 [file 12903_2024_4098_MOESM1_ESM.docx]
